# Supplementary material for: Circulating microRNAs as biomarkers for diabetic retinopathy stage identification: A DTA systematic review and meta-analysis
Source: PLoS One. 2025 Nov 21;20(11):e0335434. doi: 10.1371/journal.pone.0335434 (PMC12637958; doi:10.1371/journal.pone.0335434)
Supplement: S1 File — S1 Text. Complete search strategy. S1 Table. Prisma DTA abstract checklist. S2 Table. Prisma DTA checklist. S3 Table. Extraction data. S4 Table. Dataset used for STATA meta-analysis and meta-regression (CTL vs DR). S5 Table. Dataset used for STATA meta-analysis and meta-regression (T2DM vs DR). S6 Table. Dataset used for STATA meta-analysis and meta-regression (NPDR vs PDR). (ZIP) [file pone.0335434.s001.zip › Supporting Information/S1 Table. Prisma DTA for abstracts checklist.pdf]

PRISMA-DTA for Abstracts Checklist

Section/topic	#	PRISMA-DTA for Abstracts Checklist item	Reported on page #
**TITLE and PURPOSE**			
Title	1	Identify the report as a systematic review (+/- meta-analysis) of diagnostic test accuracy (DTA) studies.	✓ 1
Objectives	2	Indicate the research question, including components such as participants, index test, and target conditions.	✓ 1
**METHODS**			
Eligibility criteria	3	Include study characteristics used as criteria for eligibility.	✓ 1
Information sources	4	List the key databases searched and the search dates.	✓ 1
Risk of bias & applicability	5	Indicate the methods of assessing risk of bias and applicability.	✓ 1
Synthesis of results	A1	Indicate the methods for the data synthesis.	✓ 1
**RESULTS**			
Included studies	6	Indicate the number and type of included studies and the participants and relevant characteristics of the studies (including the reference standard).	✓ 1-2
Synthesis of results	7	Include the results for the analysis of diagnostic accuracy, preferably indicating the number of studies and participants. Describe test accuracy including variability; if meta-analysis was done, include summary results and confidence intervals.	✓ 1-2
**DISCUSSION**			
Strengths and limitations	9	Provide a brief summary of the strengths and limitations of the evidence	✓ 1-2*
Interpretation	10	Provide a general interpretation of the results and the important implications.	✓ 2*
**OTHER**			
Funding	11	Indicate the primary source of funding for the review.	✓ 1*
Registration	12	Provide the registration number and the registry name	? 1*

Adapted From: McInnes MDF, Moher D, Thombs BD, McGrath TA, Bossuyt PM, The PRISMA-DTA Group (2018). Preferred Reporting Items for a Systematic Review and Meta-analysis of Diagnostic Test Accuracy Studies: The PRISMA-DTA Statement. JAMA. 2018 Jan 23;319(4):388-396. doi: 10.1001/jama.2017.19163.

*Strengts such using GRADE are in methods (see abstract)
*Limitations are reflected in the conclusions (see abstract)
*Interpretation are described in conclusions (see abstract)
*Funding are described in methods (see abstract)
*Registration: The protocol was not registered, but it is defined as unregistered in the abstract.**
