# Supplementary material for: Circulating microRNAs as biomarkers for diabetic retinopathy stage identification: A DTA systematic review and meta-analysis
Source: PLoS One. 2025 Nov 21;20(11):e0335434. doi: 10.1371/journal.pone.0335434 (PMC12637958; doi:10.1371/journal.pone.0335434)
Supplement: S1 File — S1 Text. Complete search strategy. S1 Table. Prisma DTA abstract checklist. S2 Table. Prisma DTA checklist. S3 Table. Extraction data. S4 Table. Dataset used for STATA meta-analysis and meta-regression (CTL vs DR). S5 Table. Dataset used for STATA meta-analysis and meta-regression (T2DM vs DR). S6 Table. Dataset used for STATA meta-analysis and meta-regression (NPDR vs PDR). (ZIP) [file pone.0335434.s001.zip › Supporting Information/S1 Text. Complete search strategy.docx]

**SEARCH STRATEGY**

**PUBMED**

(("diabetic retinopathy"[Title/Abstract] OR "diabetic retinopathies"[Title/Abstract] OR "retinopathies, diabetic"[Title/Abstract] OR "diabetic macular edema"[Title/Abstract] OR "proliferative diabetic retinopathy"[Title/Abstract] OR "non-proliferative diabetic retinopathy"[Title/Abstract]) AND ("microRNAs"[Title/Abstract] OR "miRNA"[Title/Abstract] OR "miR"[Title/Abstract] OR "micro RNA"[Title/Abstract] OR "pri-miRNA"[Title/Abstract] OR "stRNA"[Title/Abstract] OR "circulating microRNAs"[Title/Abstract] OR "exosomal microRNAs"[Title/Abstract])) AND ("profiling"[All Fields] OR "expression"[All Fields] OR "alteration"[All Fields] OR "biomarkers"[All Fields]) AND ("humans"[MeSH Terms] OR "clinical study"[All Fields] OR "clinical trial"[All Fields] OR "case-control study"[All Fields] OR "cohort study"[All Fields]) AND ("serum"[All Fields] OR "plasma"[All Fields] OR "aqueous humor"[All Fields] OR "extracellular vesicles"[All Fields] OR "exosomes"[All Fields]) NOT ("in vitro"[All Fields] OR "animal model"[All Fields] OR "mouse model"[All Fields] OR "rat model"[All Fields] OR "rodent model"[All Fields]) AND ("2000/01/01"[Date - Publication] : "2025/01/20"[Date - Publication]))

**CENTRAL**

("diabetic retinopathy" OR "diabetic retinopathies" OR "retinopathies, diabetic" OR "diabetic macular edema" OR "proliferative diabetic retinopathy" OR "non-proliferative diabetic retinopathy") AND("microRNAs" OR "miRNA" OR "miR" OR "micro RNA" OR "pri-miRNA" OR "stRNA" OR "circulating microRNAs" OR "exosomal microRNAs")AND("profiling" OR "expression" OR "alteration" OR "biomarkers")AND ("humans" OR "clinical study" OR "clinical trial" OR "case-control study" OR "cohort study") AND ("serum" OR "plasma" OR "aqueous humor" OR "extracellular vesicles" OR "exosomes") NOT("in vitro" OR "animal model" OR "mouse model" OR "rat model" OR "rodent model")

**WEB OF SCIENCE**

TS=("diabetic retinopathy" OR "diabetic retinopathies" OR "retinopathies, diabetic" OR "diabetic macular edema" OR "proliferative diabetic retinopathy" OR "non-proliferative diabetic retinopathy")AND TS=("microRNAs" OR "miRNA" OR "miR" OR "micro RNA" OR "pri-miRNA" OR "stRNA" OR "circulating microRNAs" OR "exosomal microRNAs") AND TS=("profiling" OR "expression" OR "alteration" OR "biomarkers") AND TS=("serum" OR "plasma" OR "aqueous humor" OR "extracellular vesicles" OR "exosomes") NOT TS=("in vitro" OR "animal model" OR "mouse model" OR "rat model" OR "rodent model")

**SCOPUS**

TITLE-ABS-KEY("diabetic retinopathy" OR "diabetic retinopathies" OR "retinopathies, diabetic" OR "diabetic macular edema" OR "proliferative diabetic retinopathy" OR "non-proliferative diabetic retinopathy") AND TITLE-ABS-KEY("microRNAs" OR "miRNA" OR "miR" OR "micro RNA" OR "pri-miRNA" OR "stRNA" OR "circulating microRNAs" OR "exosomal microRNAs") AND TITLE-ABS-KEY("profiling" OR "expression" OR "alteration" OR "biomarkers") AND TITLE-ABS-KEY("serum" OR "plasma" OR "aqueous humor" OR "extracellular vesicles" OR "exosomes") AND TITLE-ABS-KEY("human" OR "clinical study" OR "clinical trial" OR "case-control study" OR "cohort study") AND PUBYEAR > 2000 AND NOT TITLE-ABS-KEY("in vitro" OR "animal model" OR "mouse model" OR "rat model" OR "rodent model")

**CLINICAL TRIAL**

("diabetic retinopathy" OR "diabetic macular edema" OR "proliferative diabetic retinopathy" OR "non-proliferative diabetic retinopathy") AND ("microRNA" OR "miRNA" OR "miR" OR "circulating microRNA" OR "exosomal microRNA") AND ("biomarker" OR "expression" OR "profiling" OR "extracellular vesicles" OR "exosomes")

**SCIENCE DIRECT**

("diabetic retinopathy" OR "diabetic macular edema" OR "proliferative diabetic retinopathy" OR "non-proliferative diabetic retinopathy") AND ("microRNA" OR "miRNA" OR "miR" OR "circulating microRNAs" OR "exosomal microRNAs") AND ("expression" OR "profiling" OR "alteration" OR "biomarkers") AND("serum" OR "plasma" OR "aqueous humor" OR "extracellular vesicles" OR "exosomes")
