# Supplementary material for: Circulating microRNAs as biomarkers for diabetic retinopathy stage identification: A DTA systematic review and meta-analysis
Source: PLoS One. 2025 Nov 21;20(11):e0335434. doi: 10.1371/journal.pone.0335434 (PMC12637958; doi:10.1371/journal.pone.0335434)
Supplement: Attachment — Submitted filename: Rebuttal Letter.docx [file pone.0335434.s003.docx]

**REBUTTAL LETTER (Reviewer 1)**

Reviewer #1: The manuscript is important and relevant topic with clear implications. The authors present a well defined question, their methods appear sound and appropriate The data are adequately analysed, and the conclusions are generally supported by the findings.

Strengths of the manuscript:

- The study addresses a significant gap in the literature and is timely.
- Methodology is robust, with appropriate controls and sample size.
- The manuscript is well written and structured logically.
- The authors have provided access to the underlying data, in compliance with the PLOS data availability policy.

Areas for improvement:

1. **While the data are available, the accompanying metadata or code used for analysis (if any) could be shared to enhance transparency and reproducibility.**

To enhance transparency and reproducibility, we have included a supplementary folder containing the raw data files used for the meta-analysis in STATA format. Recognizing that not all users may have access to STATA, we also provide the same datasets in Excel format. These datasets cover the three diagnostic comparisons performed in the review and include the essential 2×2 table data: true positives (TP), true negatives (TN), false positives (FP), and false negatives (FN), along with a matrix of probabilities ranging from 0 to 1 for use in meta-regression analyses. The STATA code used for the analyses is also provided below

| **TEST** | **STATA CODE** |
| --- | --- |
| Forest plots of sensitivity, specificity, and likelihood ratios | midas tp fp fn tn, texts(0.60) bfor(dss) id(study) ford fors |
| Summary ROC curve (SROC) | midas tp fp fn tn, sroc(both) |
| Fagan nomogram for post-test probability | midas tp fp fn tn, fagan(0.15) |
| Meta-regression to explore heterogeneity | midas tp fp fn tn, reg(variable) |
| Sensitivity analysis | midas tp fp fn tn, modchk(all) |
| Funnel plot for publication bias (Deeks' test) | midas tp fp fn tn, pubbias |

2. **In a few places, more detail regarding statistical methods or rationale for specific analytical decisions would strengthen the manuscript.**

We appreciate the reviewer’s suggestion and have revised the Statistical Analysis section to provide greater clarity and methodological detail. Specifically, we have now included a justification for the use of the bivariate random-effects model, following the Cochrane Handbook for DTA reviews (Chapter 10), and clarified that empirical SROC curves were generated from the bivariate estimates due to inconsistent reporting of diagnostic thresholds across studies.

We have also expanded on the procedures used to assess and explore heterogeneity, including the use of Cochran’s Q-test, I² statistic, and pre-specified meta-regression analyses, detailing the covariates examined (e.g., sample size, sample type, normalization strategy).

We believe that these revisions enhance the transparency and robustness of our statistical methodology.

3. **The discussion could be expanded slightly to more clearly situate the findings within the context of existing literature.**

The discussion section has been restructured to enhance clarity and depth, and the results have been systematically compared with existing literature to contextualize the findings.

4. **Please do acknowledge that the lack of protocol registration (line 74) is a limitation.**

Thank you for your observation. We have incorporated the absence of protocol registration in PROSPERO into the Discussion section and acknowledged it as a limitation of the study.

5. **Was there a need for 2x2 tables or individual results reported? I do not recall seeing these.**

In the raw data folder we have provided, you will find an Excel file for each of the three diagnostic comparisons. Each file contains the 2×2 contingency tables constructed for every individual study, already formatted for direct import into STATA. We will include these files as supplementary material to ensure full transparency and reproducibility of the analysis.

Files Name:

S5 Table. Dataset used for STATA meta-analysis and meta-regression (CTL vs DR)

S5 Table. Dataset used for STATA meta-analysis and meta-regression (T2DM vs DR)

S6 Table. Dataset used for STATA meta-analysis and meta-regression (NPDR vs PDR)

6. **Some inconsistency in abbreviations: e.g. "NPL" should be NLR (Negative Likelihood Ratio). Rather replace "NPL" with "NLR" consistently.**

The errors in this abbreviation have been corrected in the manuscript.

7. **Check for citation styles I may have picked up the use of Vancouver and APA. please choose the one required by the journal. Superscript references (e.g., "22% (1)") are inconsistently formatted.**

All references have been reviewed and formatted according to the Vancouver citation style, as required by PLOS ONE. We appreciate the comment.

8. **Please double check this. You wrote "RD" in some places instead of DR (e.g., line 268, 269, etc.). Correct “RD” to DR (Diabetic Retinopathy) to maintain consistency**.

The errors in this abbreviation have been corrected in the manuscript. We appreciate the comment.

**REBUTTAL LETTER (Reviewer 2)**

Reviewer #2: The study conducted by Martinez Santos et al performed a systemic review and meta-analysis on the different DR stages by screening countless published works. They then analyzed the 16 studies and found circulating miRNAs of interest for future diagnostic testing. The appreciate the Martinez Santos and colleagues for conducting important research in the field of diabetes. There are only a few minor suggestions prior to the manuscript being fully ready for publication.

Major:

**1.It would be nice to briefly touch upon previous efforts that have studies DR and the possible biomarkers that were identified, if any, and where they may have fallen short on to enhance the motivation behind this work. Perhaps including a brief paragraph diving into this between the paragraphs on line 60 and 61 might help with the flow**.

Thank you for this insightful suggestion. We have revised the Introduction to include a brief summary of previous studies exploring circulating miRNAs as biomarkers for DR. In particular, we note that while many individual studies and one prior meta-analysis have evaluated the diagnostic potential of miRNAs, none have stratified performance by disease stage or type of control group, highlighting a clear gap in the literature that this review aims to address.

Minor:

**1. Please provide citation for the claim of 92.6 million on line 44.**

A portion of the introduction has been revised based on suggestions from other reviewers. This specific sentence is no longer included in the current version. Nevertheless, we appreciate the comment.

**2. On line 54, specifying what types of bodily fluids miRNAs can be found would be neat for potential readers to grasp at.**

We agree with the reviewer and have now included specific examples of the biological fluids in which miRNAs are detectable. These include serum, plasma, aqueous humor, and extracellular vesicles, as indicated in the revised third paragraph of the Introduction.

**3. On line 95, was gestational diabetes also excluded?**

Thank you for your observation. Gestational diabetes was not included in the review; however, this was not clearly stated in the original manuscript. To address this, we have revised the sentence from: “We excluded studies that focused only on type 1 diabetes” to: “We excluded studies that focused on other types of diabetes, such as type 1 or gestational diabetes.” This change ensures that the eligibility criteria are clearly defined and helps avoid any potential confusion regarding the study scope.

**4. For figure 3, it would be nice to see the 45 degree dashed line on panels C, F, I to show random chance and for visualization purposes.**

We appreciate your comment and suggestion. However, after incorporating a 45-degree dividing line, the figure appears visually fragmented, as if it were two separate panels rather than one cohesive figure. In our interpretation, this disrupts the visual flow and clarity, particularly since the panel labels (A, B, C) are intended to be read sequentially. For this reason, we chose to maintain the original layout to preserve consistency and facilitate interpretation.

**5. A side question, but I noticed in the supplements that sex and age was recorded. Were there any other potential features that were recorded by any chance?**

Sex and age were indeed collected as variables to explore whether miRNA expression patterns might differ according to these demographic factors. However, due to the high degree of heterogeneity identified in our overall statistical analysis, incorporating these variables into subgroup analyses would not have yielded robust or reliable conclusions and might have introduced additional bias.

Another clinically relevant variable that we initially intended to include was blood glucose levels. While some studies reported this parameter, the inconsistency across the dataset due to missing values in several studies, led us to exclude it from the final analysis. Had this variable been consistently reported, it would have allowed us to assess correlations between glycemic control and miRNA expression, especially given that glucose level thresholds are globally standardized and clinically stratified. We agree that this remains an important area for future investigation, and we appreciate the reviewer’s curiosity on this point.

**REBUTTAL LETTER (Reviewer 3)**

Reviewer #3: Thank you for the opportunity to review the manuscript titled "microRNA-Diagnostic Test Accuracy (DTA) for diabetic retinopathy stage identification: A systematic review and meta-analysis" for consideration in PLOS ONE journal. This study aims to evaluate the diagnostic accuracy of circulating microRNAs (miRNAs) as biomarkers for distinguishing between different stages of diabetic retinopathy (DR), focusing on three specific comparisons: healthy controls versus diabetic retinopathy (CTL vs DR), type 2 diabetes mellitus without retinopathy versus diabetic retinopathy (T2DM vs DR), and non-proliferative diabetic retinopathy versus proliferative diabetic retinopathy (NPDR vs PDR).

**1. Title & Abstract**

**1.1 Title Ambiguity (Page 1, Line 1): The title does not fully reflect the study's scope. It uses "microRNA-Diagnostic Test Accuracy (DTA)" and "stage identification" but fails to specify that it evaluates circulating miRNAs across distinct DR stages.**

We thank the reviewer for this insightful suggestion. To enhance clarity and fully comply with the PRISMA-DTA guidance (Item 1) we have revised our title as follows: Circulating microRNAs as biomarkers for diabetic retinopathy stage identification: a DTA systematic review and meta-analysis.

This revised title:

1. **Specifies the specimen type** (“Circulating microRNAs”),
2. **Describes the clinical task** (“diabetic retinopathy stage identification”),
3. **Identifies the study design** (“a DTA systematic review and meta-analysis”),
4. **Remains well under** the 250-character limit recommended by PLOS ONE, and
5. **Conforms exactly** to PRISMA-DTA for Abstracts Item 1 by clearly signaling that this is a diagnostic test accuracy (DTA) review and meta-analysis.

**1.2 Inadequate Conciseness and Informativeness (Page 1, Line 1): While concise, the title omits critical details, such as the focus on circulating miRNAs and the specific staging comparisons.**

Thank you for raising the importance of balancing informativeness with brevity. In addition to PLOS ONE’s recommendation that titles be concise and clear, the PRISMA-DTA Statement (Item 1) further advises that a title should “identify the report as a systematic review and/or meta-analysis of diagnostic test accuracy studies” without becoming excessively long or unwieldy. Listing each of the three DR stages explicitly in the title would indeed convey complete specificity, but at the cost of excessive length and reduced readability.

**1.3 Abstract Non-Compliance with PRISMA (Page 1, Lines 15-39): The abstract claims adherence to PRISMA-DTA guidelines but falls short. It lacks a detailed description of the search strategy (e.g., specific terms or dates), does not specify the number of reviewers involved in all processes, and omits explicit reporting of confidence intervals for all numerical results in a consistent manner.**

We appreciate the reviewer’s careful and constructive feedback:

Search strategy: We have specified all six databases (PubMed, CENTRAL, Scopus, Web of Science, ScienceDirect, ClinicalTrials.gov) and the search cutoff date (15 January 2025). According to PRISMA-DTA for Abstracts, this level of detail fulfills the requirement for information sources and dates; detailed search terms are provided in the full methods section.

Reviewers: The abstract states “Two reviewers independently performed study selection,” which meets PRISMA-DTA standards for describing selection processes. The full Methods clarifies that discrepancies were resolved by a third reviewer, ensuring methodological rigor without overburdening the abstract.

Confidence intervals: All pooled estimates now include correctly formatted 95% confidence intervals.

**1.4 Incomplete Key Findings (Page 1, Lines 29-34): The abstract reports pooled sensitivity and specificity but does not include confidence intervals consistently (e.g., "77% (95% CI 0.070-82)" uses an unconventional format). It also omits the number of participants per comparison and specific miRNAs analyzed.**

We thank the reviewer for highlighting these points:

Confidence-interval format: We have standardized all confidence intervals to the conventional format. We have presented sensitivity and specificity values as percentages along with their confidence intervals, e.g., 77% (70–82), as these metrics are more intuitively understood in this format within the context of this type of analysis.

Participants per comparison: To maintain abstract conciseness and readability,as recommended by PLOS ONE’s author guidelines and PRISMA‑DTA, we report the total sample size (n = 1849) and total number of studies (16) in the abstract (Item 7). Providing per-contrast patient counts would exceed typical abstract length limits.

List of miRNAs: Enumerating all 21 individual miRNAs is beyond the scope of an abstract summary. We have instead clearly stated the total number of miRNAs evaluated.

Here is a side‑by‑side check of our current abstract against the 12‑item PRISMA‑DTA for Abstracts checklist, in case you wish to examine the checklist items in more detail.

**PRISMA-DTA for Abstracts**

| PRISMA-DTA item | Present in current abstract? | Gap / comment |
| --- | --- | --- |
| 1. Title identifies DTA SR/MA | ✓ Yes | Title specifies systematic review and meta-analysis of diagnostic accuracy (PRISMA-DTA Item 1). |
| 2. Objectives (Population, Index test, Target condition) | ✓ Yes | Fully defined: adults with T2DM; circulating miRNAs; DR staging. |
| 3. Eligibility criteria | ✓ Yes | Databases, date range, study designs, reference standard clearly stated. |
| 4. Information sources & dates | ✓ Yes | Six databases searched to 15 Jan 2025. |
| 5. Risk-of-bias & applicability methods | ✓ Yes | QUADAS-2 independent appraisal by two reviewers. |
| 6. Synthesis methods | ✓ Yes | Bivariate random-effects meta-analysis with subgroup, meta-regression, sensitivity analyses. |
| 7. Included studies & key characteristics | ✓ Yes | 16 studies (n=1849; 29 miRNAs). |
| 8. Quantitative results | ✓ Yes | Sens, Spec, AUC with correct 95% CIs for all three comparisons. |
| 9. Strengths & limitations | ✓ Yes | Added evidence certainty (low-moderate) and main bias concerns. |
| 10. Interpretation / implications | ✓ Yes | Clear summary of clinical relevance and future research needs. |
| 11. Funding | ✓ Yes | “No external funding” declared. |
| 12. Registration | ✓ Yes | “Protocol not registered” stated per PRISMA-DTA. |

**2. Introduction**

**2.1 Unclear Research Questions and Objectives (Page 10, Lines 42-69): The introduction states the goal of evaluating miRNA diagnostic accuracy (Line 68) but does not explicitly define the research questions or detail the three comparisons (CTL vs DR, T2DM vs DR, NPDR vs PDR) until later sections.**

Thank you for this observation. We have revised the final paragraph of the Introduction to clearly state the objective of our review and explicitly describe the three diagnostic comparisons analyzed: (1) healthy controls vs DR patients, (2) T2DM without DR vs with DR, and (3) NPDR vs PDR. We believe this clarification improves the focus and structure of the introduction.

**2.2 Insufficient Background (Page 10, Lines 43-52): The background mentions DR prevalence and diagnostic tool limitations but lacks detail on the clinical significance of staging DR (e.g., NPDR vs PDR progression).**

We appreciate this suggestion and have expanded the first paragraph of the Introduction to explain the clinical relevance of DR staging. Specifically, we now mention that progression from NPDR to PDR increases the risk of vision-threatening complications and guides therapeutic decisions, thereby underscoring the importance of accurate stage-specific diagnosis.

**2.3 Weak Rationale (Page 10, Lines 61-69): The rationale for this meta-analysis is underdeveloped. It notes the need for minimally invasive methods (Line 51) but does not explain why existing miRNA studies are insufficient or how this review fills a specific gap.**

We thank the reviewer for this observation. In response, we would like to highlight that the Introduction now includes a clear rationale supported by two key points. First, it states that while numerous studies have evaluated the diagnostic potential of circulating miRNAs in DR, there is marked heterogeneity in sample types, analytical methods (e.g., RT-qPCR, microarrays, NGS), and target miRNAs.

**2.4 Undefined Knowledge Gap (Page 10, Lines 61-63): The introduction hints at a lack of "comprehensive evaluations" but does not clearly articulate the specific knowledge gap this study addresses, such as inconsistencies in prior miRNA research or unmet clinical needs.**

We would like to clarify that the revised Introduction now explicitly outlines the knowledge gap that this review addresses. In particular, we state that although many individual studies,and one prior meta-analysis,have explored the diagnostic value of circulating miRNAs in DR, no previous work has performed a stratified meta-analysis in this field.

**3. Methods**

**3.1 Poorly Defined Eligibility Criteria (Page 12, Lines 84-96): The criteria mention human participants and fundus fluorescein angiography as the gold standard but lack specificity on DR stage definitions, miRNA types (e.g., single vs. panels), or exclusion of studies with incomplete data**.

We have revised the Eligibility Criteria section to clarify several key aspects. First, we now specify that both fundus examination and fluorescein angiography were accepted as reference standards for classifying DR, in accordance with the methods reported in the included studies.

Second, we have explicitly mentioned that DR was assessed at different stages, including non-proliferative (NPDR) and proliferative (PDR) forms, to provide greater specificity regarding the disease spectrum.

Finally, while we did not provide an exhaustive list of all exclusion reasons to preserve brevity, we believe the criteria explicitly listed, such as the exclusion of studies focused solely on type 1 diabetes, animal models, or *in vitro* data, are sufficient to convey the scope and intent of our selection process. The inclusion and exclusion criteria are well defined and consistent with the aims and structure of a diagnostic accuracy meta-analysis, as per PRISMA-DTA recommendations.

**3.2 Vague Study Selection and Data Extraction (Page 12, Lines 97-103): The process mentions two reviewers and a third for arbitration but lacks detail on how disagreements were resolved (e.g., consensus or voting) or what specific data were extracted (e.g., miRNA thresholds).**

We appreciate the reviewer’s suggestion to clarify the study selection and data extraction process. In response, we have revised the *Study Selection and Data Extraction* section to specify that discrepancies between reviewers were resolved by consensus, with a third reviewer providing arbitration when necessary.

We also expanded the description of the extracted data to include: diagnostic accuracy metrics (sensitivity, specificity, AUC, PLR, NLR, DOR), miRNA expression trends, and cut-off values when available, as we acknowledge that these were not consistently reported across all included studies.

**3.3 Inadequate Risk of Bias Tool Application (Page 13, Lines 105-109): The use of QUADAS-2 is noted, but there’s no explanation of how it was applied (e.g., domain-specific scoring) or how results influenced the analysis.**

Thank you for this observation. We have revised the *Quality Assessment* section to clarify the application of the QUADAS-2 tool. Specifically, we now state that it was applied independently by two reviewers across the four standard domains (patient selection, index test, reference standard, and flow/timing), and that discrepancies were resolved by consensus.

In addition, we explain that the QUADAS-2 assessments informed the summary of risk of bias and were considered in the interpretation of our findings, as well as in the GRADE-based assessment of overall evidence certainty.

**3.4 Unclear Statistical Methods (Page 13, Lines 110-125): The methods mention a bivariate random-effects model and heterogeneity tests but do not justify the model choice, detail heterogeneity exploration beyond, or explain meta-regression variables.**

Thank you for your observation. We have revised the *Statistical Analysis* section to explicitly justify the use of the bivariate random-effects model, which is recommended for diagnostic test accuracy meta-analyses as it jointly estimates sensitivity and specificity while accounting for their correlation and inter-study variability (Cochrane Handbook DTA, Chapter 10).

<https://training.cochrane.org/handbook-diagnostic-test-accuracy/2023-07/chapter-10>

Furthermore, we now clearly state that empirical SROC curves were generated from the bivariate estimates since diagnostic thresholds were not consistently reported across studies, making a hierarchical HSROC model inappropriate withour cut off values.

We have also clarified that heterogeneity was assessed using Cochran’s Q and I² derived from the bivariate model, and that pre-specified univariable meta-regressions were performed to explore sources of heterogeneity. The covariates tested include sample size, country of origin, biological specimen type, normalization strategy, and miRNA expression pattern.

We believe these revisions address the reviewer’s concerns and improve the clarity and methodological transparency of our analysis.

**4. Results**

**4.1 Opaque PRISMA Flow Diagram (Page 14, Lines 127-136): The flow diagram (Figure 1) is referenced, but exclusions like "objective different from the study"**

Thank you for your valuable observation regarding the phrasing "objective different from the study" in the PRISMA flow diagram (Figure 1). We acknowledge that the original wording was too general and may have lacked clarity.

In response, we have revised this category to "Microvascular complications other than DR" to more accurately reflect the nature of the excluded studies. This change refers to full-text articles that focused on other diabetes-related microvascular complications such as diabetic nephropathy, neuropathy, or macrovascular events, rather than on the diagnostic accuracy of circulating miRNAs for diabetic retinopathy. These studies, while relevant in a broader diabetic context, did not meet our predefined inclusion criteria.

We appreciate your suggestion, which helped us improve the transparency and precision of the flow diagram

**4.2 Incomplete Study Characteristics (Page 15, Line 155; Table 1): Table 1 lists studies but omits critical details, such as specific miRNAs per study or diagnostic thresholds.**

Table 1 explicitly includes a dedicated column for the specific miRNAs analyzed in each study (5th column, titled “miRNAs”), where each relevant molecule is clearly listed per study.

Regarding diagnostic thresholds (cut-off values), we acknowledge that not all included studies reported this parameter. While a few studies did provide cut-offs, the lack of uniform reporting precluded their integration into the pooled statistical analysis. This issue is common in diagnostic meta-analyses of miRNA biomarkers. For instance, in the only other meta-analysis in this area (Ma et al., Front Endocrinol, 2022;13:929924), cut-offs were reported inconsistently and were likely not used in calculating pooled metrics.

We have been transparent about this limitation and have explicitly addressed it in the revised Discussion section with the following sentence: “Finally, most studies did not consistently define diagnostic cut-off values, prompting us to adopt a hierarchical SROC modeling approach rather than synthesizing data at a single threshold. In fact, the cut-off value data were collected and are included in Annex 1, which was submitted during the revision and is now titled **S3 Table. Extraction data**, to further ensure transparency.

We believe this approach ensures methodological soundness while reflecting the reality of the available evidence.

**4.3 Unjustified Risk of Bias Reporting (Page 16, Lines 156-167): The risk of bias summary (Figure 2) notes patient selection concerns but lacks specific examples or impact analysis, undermining its justification and usefulness.**

We respectfully clarify that the QUADAS‑2 tool is the internationally endorsed method for assessing risk of bias in diagnostic test accuracy studies, as recommended by the PRISMA-DTA statement and the Cochrane Handbook for Systematic Reviews of DTA.

The judgments presented in Figure 2 are the result of a structured, domain-based evaluation conducted by two independent reviewers, as outlined in the Methods (Section 3.1). In the manuscript, risk of bias in patient selection is explicitly acknowledged in the Results and Discussion, and was mainly attributed to the use of retrospective and case-control designs, which is reported clearly in the “Study characteristics and quality assessment” section.

While we understand the reviewer’s request for more detailed examples, the goal of the QUADAS‑2 summary figure is to provide a visual overview of bias across studies. The relevant methodological limitations and their implications are already discussed in the text. Specifically, the Discussion highlights patient selection bias as a key factor that may have influenced the certainty of evidence.

We would also like to note that QUADAS‑2 is designed not only to identify bias, but to highlight recurring design limitations in the field. Even when studies do not meet the highest methodological standards, this structured evaluation helps define where and how future research can improve, particularly in terms of prospective designs, recruitment methods, and clearer reporting.

We hope this clarification reinforces the value and justification of our bias assessment.

**4.4 Weak Meta-Analysis Reporting (Page 16-17, Lines 172-218): Results report sensitivity and specificity but inconsistently present confidence intervals (e.g., "77% (95% CI: 0.70-0.82)" vs. "84% (79-87)"). High heterogeneity is noted but not adequately explored or mitigated, questioning robustness.**

Thank you for this observation. We have now standardized the reporting of diagnostic metrics throughout the manuscript as we mentioned above.

Regarding heterogeneity, we respectfully believe this concern may arise from a misinterpretation of our analytical strategy. In our study, heterogeneity is not ignored, on the contrary, it is thoroughly explored and transparently reported through several complementary approaches:

In Section 3.3 (Subgroup Analysis and Meta-Regression), we systematically investigated the impact of key covariates, such as matrix type, normalization strategy, expression trend, and geographic origin, on pooled diagnostic performance.

In Section 3.4 (Sensitivity Analysis), we used Cook’s distance and standardized residuals to identify potential influential studies. Their exclusion produced only marginal shifts in pooled estimates and did not significantly alter heterogeneity metrics (I²), confirming the stability and reliability of the overall results.

We would like to underscore that heterogeneity is expected in meta-analyses of diagnostic biomarkers, particularly those covering diverse populations and methodologies. The presence of heterogeneity does not inherently weaken a meta-analysis, what matters is whether it is identified, explored, and transparently discussed, as we have done following PRISMA-DTA and Cochrane Handbook guidance.

Therefore, we disagree with the suggestion that our findings lack robustness. On the contrary, the combination of structured subgroup analyses, meta-regressions, and sensitivity testing demonstrates that our results are both methodologically sound and reproducible. Moreover, acknowledging methodological limitations and variation strengthens scientific credibility and helps define the path forward for higher-quality future studies.

**5. Discussion**

**5.1 Limited Contextualization (Page 24, Lines 326-340): The discussion references Ma et al. (2022) but does not deeply compare findings or explain how this study advances prior work.**

Thank you for this important observation. We have now substantially deepened the comparison with Ma et al. (2022) in the revised Discussion). Specifically, while Ma et al. performed a well-conducted meta-analysis, their approach aggregated diabetic and healthy controls into a single reference group and analyzed all DR stages together. This likely increased clinical heterogeneity and limited the translational utility of their pooled estimates. In contrast, our study advances the field by introducing a stratified meta-analytic framework based on three specific clinical comparisons (CTL vs DR, T2DM vs DR, and NPDR vs PDR), enabling a more nuanced evaluation of diagnostic performance across disease stages. Moreover, unlike Ma et al., we applied the GRADE approach to formally assess the quality of evidence, which enhances transparency and interpretability for clinicians and policymakers.

**5.2 Inadequate Limitation Discussion (Page 25, Lines 369-384): Limitations mention demographic bias and study design but overlook key issues like variability in miRNA detection methods or lack of standardized thresholds.**

We agree with the reviewer and have addressed this in the revised Limitations section. In particular, we now discuss the lack of standardized diagnostic cut-off values across studies and the impact of this omission on clinical applicability. We also describe the methodological heterogeneity in sample processing (plasma vs. serum), normalization strategies (e.g., U6 vs miR-16), and detection platforms, all of which contribute to inconsistency in diagnostic performance. These additions strengthen our critical appraisal of the current literature.

**5.3 Unclear Implications (Page 25, Lines 386-394): Clinical and research implications are vague (e.g., "integrated with other clinical assessments" lacks specificity).**

We have revised the final paragraph of the discussion to clarify the clinical and research implications of our findings. We also call for international standardization of pre-analytical protocols, normalization strategies, and threshold definitions to ensure successful clinical translation

**6. Conclusion**

**6.1 Unjustified Claims (Page 26, Lines 387-390): The conclusion asserts "high diagnostic accuracy" despite moderate-to-high heterogeneity and low-to-moderate evidence quality (Page 22, Lines 316-318).**

We thank the reviewer for this observation and the opportunity to clarify. Upon careful review, we acknowledge that while the final conclusion section of the manuscript does not explicitly state “high diagnostic accuracy,” a more assertive wording appeared in the Abstract, which may have led to misunderstanding. In response, we have revised the Abstract to ensure alignment with the overall tone and evidence strength of the manuscript.

We would also like to respectfully point out that the revised statement emphasizes that “circulating miRNAs exhibit promising diagnostic accuracy, highlighting their potential as practical, non-invasive biomarkers,” which we believe is a more appropriate and conservative formulation that reflects the encouraging but still preliminary nature of our findings.

We fully acknowledge moderate-to-high heterogeneity and address it explicitly:

Our stratified approach was designed precisely to reduce heterogeneity. By contrast, Ma et al. (2022) [PMID: 35898469], who performed a non-stratified analysis of circulating miRNAs for DR detection in 52 studies (n=1987 DR, 1771 controls), reported significantly higher heterogeneity as you can aprreciate in the table and compare all types of RD.

The lower I² values we report, compared to Ma et al., demonstrate that our methodology achieved greater consistency across estimates.

| **Comparison** | **Sensitivity I²** | **Specificity I²** |
| --- | --- | --- |
| **CTL vs DR** | 47.1% | 62.3% |
| **T2DM vs DR** | 73.1% | 73.1% |
| **NPDR vs PDR** | 55.6% | 77.1% |
| **Ma et al. (2022)** | **85.1%** | **77.0%** |

By stratifying comparisons and exploring heterogeneity via subgroup and meta-regression analyses, we were able to refine our estimates and demonstrate their relative stability compared to broader meta-analyses. The result supports the cautious optimism reflected in our conclusion. We also clearly recommend further large-scale, prospective studies in diverse populations to confirm these results before clinical implementation in conclusion section.

**6.2 Lack of Specificity (Page 26, Lines 391-394): It calls for future studies but does not specify research directions (e.g., which miRNAs or methods).**

We appreciate your insightful comment. To avoid overextending the conclusion section, we have expanded the suggestions for future research directions within the discussion. Specifically, we have added examples of relevant miRNAs and methodological approaches that warrant further investigation, in line with your recommendation.

**7. Transparency & Reproducibility**

**7.1 Unregistered Study (Page 11, Line 73): The lack of registration (e.g., PROSPERO) deviates from best practices and reduces transparency.**

We acknowledge this point. While protocol registration is not a formal requirement, we recognize that it contributes to greater transparency and reproducibility. We will definitely consider registering the protocol in future studies to strengthen methodological rigor.

**REBUTTAL LETTER (Reviewer 4)**

1) **Grammatical and Orthographic Comments**

**1.1 Consistency: The manuscript sometimes switches between percentage formats (e.g., “77 %” and “77%”). Choose one and apply it uniformly (journal style guides typically prefer “77%” without a space).**

Thank you for the comment. The formatting of all percentage values in the manuscript has been reviewed and standardized accordingly.

**1.2 Some long sentences in the Introduction (lines 43–51) could be broken for clarity; e.g., “This makes them less accessible, especially in remote or low-resource areas.” could be split for better readability.**

The introduction has been thoroughly restructured, with the removal of lengthy sentences to enhance clarity and conciseness.

**1.3 Minor repetition: In lines 55–60, there is redundancy explaining miRNAs’ importance; consider condensing to maintain focus.**

Thank you for the comment. This sentence has been removed accordingly.

**1.4 Typo-like inconsistency: lines like “overall evidence certainly was graded via GRADE” (line 26) sound off. Perhaps intended “certainty was graded”; a correction improves precision.**

The abstract has been revised to state “overall evidence certainty was graded via GRADE.” Thank you for the valuable comment.

2) **Scientific Rigor**

**2.1 Protocol registration: The authors state the protocol was not registered. While not mandatory, protocol registration (e.g., PROSPERO) increases transparency.**

We are fully aware of this. Although protocol registration is not mandatory, doing so would enhance the reproducibility of our study. We will certainly take this into consideration for future research.

**2.2 Risk of bias: High risk in patient selection and absence of randomized trials limits robustness; the authors acknowledge this but could suggest solutions.**

We thank the reviewer for highlighting these important methodological considerations. The observed risk of bias in patient selection and the lack of randomized controlled trials (RCTs) in our analysis indeed limit the confidence in our findings. To address this, we propose the following contextual explanations and constructive recommendation.

- Practical and ethical constraints:

DTA studies often rely on observational cohort or case-control designs due to feasibility, cost, and the need to evaluate real-world diagnostic performance.As noted by Rutjes1 et al (1)., “only a small number of studies of diagnostic accuracy fulfil essential methodologic standards”. This limitation is inherent in the field and not unique to our review.

1-Rutjes AW, Reitsma JB, Di Nisio M, Smidt N, van Rijn JC, Bossuyt PM. Evidence of bias and variation in diagnostic accuracy studies. CMAJ. 2006 Feb 14;174(4):469-76. doi: 10.1503/cmaj.050090. PMID: 16477057; PMCID: PMC1373751

- Methodological sophistication of retrospective studies:

Many existing studies provide individual-level data (TP, FP, FN, TN) and report sensitivity and specificity, even if they do not follow ideal prospective designs. However, the quality of reporting in DTA research remains suboptimal, and incomplete or inconsistent statistical reporting is a documented concern

**Proposed solutions**

- **Promote comprehensive reporting:**
  Better adherence to reporting standards such as STARD and QUADAS-2 would ensure that raw data are readily available for secondary analysis.
- **Invest in training and capacity building:**
  A common barrier is the lack of methodological training among clinical researchers in diagnostic accuracy methods. Enhanced education on study design, reporting, and sample size justification would increase the quality and interpretability of DTA studies, as noted in Macaskill et al (2). and Korevaar et al (3).
- **Facilitate prospective, multicenter cohort studies:**
  Coordinated efforts to **conduct large, prospective cohorts across** various populations would reduce selection bias and enhance external validity. Such studies can yield robust estimates of diagnostic accuracy that are more resistant to bias than small, retrospective single-center studies.

2-Macaskill P, Gatsonis C, Deeks JJ, Harbord RM, Takwoingi Y. Chapter 10: Analysing and Presenting Results. In: Deeks JJ, Bossuyt PM, Gatsonis C, editors. *Cochrane Handbook for Systematic Reviews of Diagnostic Test Accuracy*. Version 1.0. Chichester (UK): John Wiley & Sons; 2010

3-Korevaar DA, Spijker R, Dinnes J, Reitsma JB, Bossuyt PM, Leeflang MM.
Reporting quality of diagnostic accuracy studies: a systematic review and meta-analysis. *Evid Based Med.* 2014;19(2):47–54.

**2.3 Applicability: Most data from Chinese cohorts; limits generalizability to other ethnicities. Consider highlighting this limitation more prominently.**

This has been addressed in Section 4. Additionally, it is discussed as a limitation in the Discussion section.

**2.4 Diagnostic thresholds: Lack of consistent cut-offs across studies weakens translation to clinical practice.**

We greatly appreciate this important observation, which addresses a key challenge in the clinical applicability of diagnostic biomarkers. The lack of consistent cut-off value across studies indeed undermines the standardization needed for real-world implementation.

In our revised submission, we have included all available cut-off values extracted from the original articles in the supplementary file, now titled S3 Table. Extraction data, to improve transparency. However, as rightly pointed out, many studies failed to report diagnostic thresholds, reflecting a common and concerning gap in the reporting of diagnostic test accuracy studies.

Despite this limitation, it is worth noting that the bivariate random-effects model we employed is designed to account for such variability. It allows for reliable estimation of pooled sensitivity and specificity even when specific thresholds are missing, by modeling the joint distribution of these two measures and incorporating between-study heterogeneity.

We have addressed this issue explicitly in the Discussion section, as we believe it represents a broader call to action: diagnostic studies should systematically report cut-off values, not only sensitivity, specificity, and sample size. The absence of threshold reporting may stem from a lack of awareness regarding its importance in the clinical translation of biomarkers. By highlighting this in both our analysis and discussion, we aim to promote more rigorous and complete reporting standards in future research.

**2.5 Some references (e.g., [33]) are relevant but recent studies post-2022 could enrich the context.**

Thank you for the suggestion; it has been taken into account during the restructuring of the Discussion section**.**

3) **Scientific Impact**

**The study fills an important gap by stratifying meta-analysis across clinically distinct DR stages, which previous works have not done in detail. This approach gives clinicians better guidance on miRNAs’ diagnostic utility at specific DR stages. The work will interest endocrinologists, ophthalmologists, and biomarker researchers; potential for real-world impact if validated prospectively.**

We sincerely thank the reviewer for this thoughtful and encouraging comment.

4) **Questions to Authors**

**4.1 Given the predominance of Chinese populations, how do you propose validating these findings across ethnically diverse cohorts?**

We appreciate this important observation regarding the demographic representation in our meta-analysis. As correctly noted, most of the included studies were conducted in Chinese populations, and we fully acknowledge this as a key limitation. In fact, this concern is explicitly addressed in both the results and discussion sections.

To mitigate this, we have incorporated subgroup and meta-regression analyses that examined the impact of study location (country of origin) on diagnostic performance. Interestingly, the data showed that diagnostic accuracy tended to be higher in studies conducted in China. While this suggests potential regional variability, it also highlights the urgent need for future diagnostic accuracy studies in more ethnically and geographically diverse cohorts.

We strongly advocate for prospective, multicenter studies involving Western and underrepresented populations. Given that glucose metabolism, gene expression patterns, and miRNA profiles can differ across ethnicities.

**4.2 How do differences in miRNA normalization strategies (e.g., U6 vs miR-16-5p) influence diagnostic performance in your dataset?**

Thank you for this insightful query. The choice of normalization strategy in circulating miRNA studies can profoundly affect quantitative results and diagnostic accuracy. Several studies have highlighted these effects:

**Implications for our meta-analysis**

In the studies we included, some used U6, others miR‑16‑5p etc. A few applied global mean or combined normalizers. Our meta-regression found that normalization method significantly contributed to heterogeneity in sensitivity and specificity estimates. Specifically, studies using U6 exhibited greater variance and slightly lower diagnostic accuracy compared to those using miR‑16‑5p or global mean approaches.

**Moving forward: recommendations**

- Standardization is critical. We recommend future diagnostic studies adopt evidence-backed strategies, such as multiple stable endogenous controls or global mean normalization, ideally validated within their specific population.
- Reporting transparency: Each study should report raw Ct values and references used for normalization, allowing meta-analysts to account for methodological variability.
- Sensitivity analyses: Where possible, replicate miRNA panels normalized via alternative references (e.g., U6 vs miR‑16‑5p) to assess impact on diagnostic performance.

**4.3 Can you discuss practical steps to standardize pre-analytical miRNA handling and measurement to improve reproducibility?**

The section of the Discussion addressing pre-analytical standardization has been expanded, as it was not previously developed in sufficient detail**.**

**4.4 Since your sensitivity analysis shows individual studies’ influence, how confident are you that these outliers don’t bias the conclusions?**

We thank the reviewer for this pertinent question. Sensitivity analysis was a key component of our approach to evaluating the robustness of our findings across all three comparisons.

Across the datasets, although certain studies (e.g., Jiang et al., Shaker et al., and Saleh et al.) were identified as influential based on Cook’s distance, their exclusion resulted in only marginal changes in pooled sensitivity and specificity, always within a range of 1–4%. These shifts were not substantial enough to affect the clinical interpretation or statistical significance of the pooled estimates.

Importantly, heterogeneity (I²) remained stable in two of the three comparisons (CTL vs DR and NPDR vs PDR), and only increased notably in the T2DM vs DR group upon removal of two studies. However, in that case, the rise in I² from 74% to 97% suggests that the influential studies may have contributed to reducing unexplained variability, rather than introducing spurious effects.

Additionally, no significant outliers were detected via standardized residuals in two of the three groups. In the NPDR vs PDR group, while two studies (Shaker et al. and Saleh et al.) exceeded the ±2 threshold, their exclusion did not substantially affect the pooled diagnostic performance, and model fit and bivariate normality remained satisfactory throughout.

Taken together, these findings provide reassurance that our conclusions are not disproportionately driven by a small number of studies. Instead, they suggest that the diagnostic performance of circulating miRNAs remains consistent and robust across a diverse set of studies and analytical conditions.

5) **Additional Comments**

**5.1 Figures and tables are well presented; figures showing forest plots, SROC curves, Fagan’s nomograms, and meta-regressions are clear and informative.**

We sincerely thank the reviewer for this positive feedback. We are pleased to know that the figures and tables were clear and informative.

**5.2 The inclusion of a detailed GRADE assessment is commendable, but the presentation of certainty ratings (Tables 5–7) could benefit from more narrative discussion in the Results or Discussion.**

We appreciate your suggestion, however, we are facing strict space limitations, as this manuscript presents three separate small-scale diagnostic meta-analyses within a single article. As you may appreciate, this format is relatively uncommon and already renders the manuscript considerably dense. Expanding the narrative discussion of GRADE assessments in detail would limit our ability to respond to other key reviewer suggestions,such as expanding the statistical analysis and enriching the discussion section, both of which we consider critical to the overall value of the manuscript.

That said, we have taken your comment seriously and made concise narrative additions to the Results and Discussion sections, summarizing the certainty levels and rationale (e.g., “The certainty of evidence for specificity was downgraded due to imprecision and suspected publication bias.”). These additions are consistent with GRADE Handbook guidance (Chapter 4.3) and the Cochrane Handbook (Chapter 14.1), both of which recommend that Summary of Findings (SoF) tables serve as the primary method for reporting certainty levels, while accompanying narrative should remain focused and interpretive, rather than duplicating the content of the tables. We believe this strikes the right balance between clarity, scientific rigor, and brevity.

<https://training.cochrane.org/handbook/current>

<https://gdt.gradepro.org/app/handbook/handbook.html>

**5.3 The Discussion excellently compares results with prior meta-analyses but could expand on how novel your subgroup stratification truly is compared to Ma et al. (2022).**

Thank you for the suggestion. We have taken this comment into account when revising the Discussion section.

**Overall Recommendation:**

The manuscript demonstrates rigorous methods and meaningful results but should address generalizability limitations more explicitly and discuss how preanalytical standardization could improve clinical adoption. Minor editorial corrections on consistency and sentence clarity are recommend
