# Supplementary material for: Circulating microRNAs as biomarkers for diabetic retinopathy stage identification: A DTA systematic review and meta-analysis
Source: PLoS One. 2025 Nov 21;20(11):e0335434. doi: 10.1371/journal.pone.0335434 (PMC12637958; doi:10.1371/journal.pone.0335434)
Supplement: Attachment — Submitted filename: Rebuttal Letter- Reviewer 5.docx [file pone.0335434.s004.docx]

**REBUTTAL LETTER (Reviewer 5)**

**Reviewer #5**: In general, the manuscript is well written and scientifically sound. The authors thoroughly revised the manuscript to address the insightful comments from the reviewers and deepened the discussion raised by them. In addition, I appreciate the effort of the authors in providing the raw data in the Excel format and the STATA code used for the analyses, which is in line with contemporary best practices that promote transparency and reproducibility. Nevertheless, there are inconsistencies that need to be resolved in order to further improve clarity and precision.

1. **Abstract section (pdf file, revised manuscript, pages 53-54)**
a) **First sentence (lines 17-18): To improve clarity and specificity, in addition to avoid redundancy (“This systematic review and meta-analysis”), I suggest the purpose of the study be modified to: “To evaluate the diagnostic accuracy of circulating miRNAs in distinguishing between different DR stages in type 2 diabetes mellitus.”**

We thank the reviewer for this helpful suggestion. We have revised the first sentence of the Abstract accordingly to improve clarity and avoid redundancy. The updated sentence now reads: “Purpose: To evaluate the diagnostic accuracy of circulating miRNAs in distinguishing between different diabetic retinopathy stages in type 2 diabetes mellitus.

b) **On line 20, it is stated that that “no external funding was received”. However, in the submission form and in the “Funding information” (pages 79-80), the authors report that “The present work received internal funds from Centro de Investigación Traslacional San Alberto Magno (CITSAM, UCV) and external funds from Agencia Estatal de Investigación Española (PID2020-117875GB-10), Instituto de Salud Carlos III (ISCIII, PI21/00083) and the European Union research fund, HORIZON MSCA 2021-DN-01-01_RETORNA 101073316 and Generalitat Valenciana ACIF 2023-128-001.” Which statement is correct?**

We sincerely thank the reviewer for pointing out this inconsistency. The projects listed indeed provide funding support to our research group; however, no funds from these grants were specifically allocated or used for the conduct of this systematic review and meta-analysis. To avoid any possible misunderstanding, we have removed the Funding section from the manuscript and clarified that this work received no dedicated external financial support.

2. **Introduction section (pdf file, revised manuscript, page 55): The first sentence (page 55, lines 73-74) states that “Diabetic retinopathy (DR) is one of the leading causes of vision loss among working-age adults worldwide (1).” That is true. However, this claim is supported by the paper by Lundeen et al. (Prevalence of Diabetic Retinopathy in the US in 2021. JAMA ophthalmology. 2023;141(8):747-54), and US is not “worldwide.**

We thank the reviewer for this accurate observation. You are correct that Lundeen et al. refers exclusively to US data and therefore does not support the global statement. This was an error on our part during referencing. The appropriate citation is RL Thomas et al. (Diabetes Res Clin Pract 2019)*, which provides worldwide prevalence data and clearly establishes diabetic retinopathy as a leading cause of vision loss among working-age adults. We have corrected the reference in the revised manuscript accordingly.

* Thomas RL, Halim S, Gurudas S, Sivaprasad S, Owens DR. IDF Diabetes Atlas: A review of studies utilising retinal photography on the global prevalence of diabetes related retinopathy between 2015 and 2018. Diabetes Res Clin Pract. 2019 Nov;157:107840. doi: 10.1016/j.diabres.2019.107840. Epub 2019 Nov 14. PMID: 31733978.

3. **Introduction section (pdf file, revised manuscript, pages 55-57): Reviewer 3 states in comment #2.3: “The rationale for this meta-analysis is underdeveloped. It notes the need for minimally invasive methods (Line 51) but does not explain why existing miRNA studies are insufficient or how this review fills a specific gap.” In the rebuttal letter, the authors responded that “In response, we would like to highlight that the Introduction now includes a clear rationale supported by two key points. First, it states that while numerous studies have evaluated the diagnostic potential of circulating miRNAs in DR, there is marked heterogeneity in sample types, analytical methods (e.g., RT-qPCR, microarrays, NGS), and target miRNAs.**

**a) I did not find this statement in the introduction of the revised manuscript.
b) What is the second key point supporting a clear rationale?**

We appreciate this helpful comment. The revised Introduction now makes the two pillars of our rationale explicit:

1.	Heterogeneity in prior studies. We have rephrased the opening sentence to state explicitly the sources of heterogeneity:

*“Although numerous studies have investigated the diagnostic potential of circulating miRNAs in DR, marked heterogeneity in sample types, analytical methods (e.g., RT-qPCR, microarrays, NGS), and target miRNAs has limited comparability across studies (14–18).”*

2.	Methodological gap in the prior meta-analysis (this is the second key point). The following sentence in the Introduction articulates the second point of our rationale:

*“Notably, one prior meta-analysis has reviewed the use of circulating miRNAs for DR detection (19), it did not perform any stratification by disease stage or type of control group. This represents a notable gap in the current literature, as the ability to distinguish early from advanced stages of DR is critical for clinical decision-making.”*

This lack of stratification mixes distinct control populations (e.g., healthy individuals together with T2DM without DR) and collapses different DR stages, which can inflate between-study heterogeneity (spectrum effects, non-comparable baselines) and reduce stage-specific interpretability of pooled diagnostic accuracy. Our review directly addresses this by pre-specifying three clinically aligned comparisons (CTL vs DR; T2DM vs DR; NPDR vs PDR), thereby improving comparability and clinical relevance of the estimates.

To keep the Introduction concise, the methodological implications of the prior meta-analysis and how our stratified design mitigates these issues are discussed in detail in the Discussion (section “comparison with previous meta-analyses”). We hope this clarification makes the two key points readily identifiable in the revised Introduction.

4. **Methods section, “Eligibility criteria” subsection (pdf file, revised manuscript, page 58, lines 137-138):Reviewer 2 asked in comment #3: “Was gestational diabetes also excluded?” In the rebuttal letter, the authors responded that they have revised the sentence from: “We excluded studies that focused only on type 1 diabetes” to: “We excluded studies that focused on other types of diabetes, such as type 1 or gestational diabetes.” This is implied in the revised sentence but not explicitly stated.**

We thank the reviewer for this observation. We confirm that gestational diabetes was excluded from this review. In the eligibility criteria we used the umbrella term “other types of diabetes” deliberately to encompass type 1 diabetes and gestational diabetes, as well as monogenic, syndromic, and secondary forms (e.g., MODY, steroid-induced diabetes, Donohue syndrome, Rabson–Mendenhall), without generating an unnecessarily long and potentially incomplete list within the Methods section. Our intention was to keep the criteria concise yet comprehensive.

5. **Results section (pdf file, revised manuscript, page 63): Table 1 is detailed and informative. However, I suggest adding a column with the total number of individuals included in each study. If necessary, due to space restrictions, the “Method” column can be removed because this information is already in the text.**

We thank the reviewer for this constructive suggestion. In line with PRISMA-DTA guidance to report participant numbers clearly, we have updated Table 1 to include an explicit “Total (N)” column (computed as TP + TN + FP + FN for each study). To preserve readability and layout, we have removed the per-study AUC column from the main table; all per-study AUC values are now provided in the Supplementary “raw dataset” table, alongside the corresponding 2×2 data. These formatting changes do not affect any pooled estimates (SROC/AUC) or conclusions reported in the manuscript.

Rationale for removing AUC (per study) rather than “Method”: The per-study AUC is often not directly comparable across studies because many papers report single-threshold accuracy (one sensitivity/specificity pair) rather than a full ROC, and AUCs may be estimated under differing assumptions. Since our quantitative synthesis relies on the bivariate model of sensitivity and specificity and presents the pooled SROC/AUC, listing per-study AUCs in the main table is redundant and can be misleading with heterogeneous thresholds. By contrast, “Method” (e.g., RT-qPCR, microarrays, NGS) describes the index test characteristics, which are essential for assessing applicability and sources of heterogeneity per PRISMA-DTA/Cochrane DTA guidance.

Methodological differences in the index test were pre-specified and used in our subgroup analyses and meta-regressions; removing this column would hinder interpretability and reproducibility for readers.

For these reasons, we believe adding Total (N) and keeping Method in the main table—while relocating per-study AUCs to the Supplement—offers the clearest and most standards-aligned presentation.

6. **Results section, “Subgroup Analysis and Meta-Regression in CTL vs DR, T2DM vs DR, and NPDR vs PDR Comparisons” subsection (pdf file, revised manuscript, page 66, lines 308-309): The authors assert that “studies conducted in China demonstrated higher diagnostic performance across all three comparison groups, as reflected by AUC.” I do not know if this is the case for CTL vs DR and T2DM vs DR. In the comparison between CTL and DR (Table 2), only one study was not performed in China, which hinders its interpretation. Regarding the comparison between T2DM and DR (Table 3), the difference in the AUC value was 0.01.**

We thank the reviewer for this careful observation. In the Results section our intention was descriptive. The subgroup AUCs are:

•	CTL vs DR: China 0.86 vs non-China 0.56

•	T2DM vs DR: China 0.87 vs non-China 0.86 (ΔAUC = 0.01)

•	NPDR vs PDR: China 0.90 vs non-China 0.87

We agree that interpretation is limited by the geographical imbalance, particularly in CTL vs DR, where only one study was conducted outside China, and that for T2DM vs DR the AUCs are essentially comparable.

This limitation is also explicitly acknowledged in the Discussion, where we note that the predominance of Chinese cohorts may introduce demographic bias and limit generalizability to other populations, underscoring the need for broader geographic representation in future studies:

*“A predominant number of the included studies were conducted in Chinese populations, which may introduce demographic bias and limit the generalizability of the findings to other ethnic groups, particularly Western cohorts. While this does not compromise internal validity, it underscores the need for broader geographic representation in future studies.”*

7. **Results section (pdf file, revised manuscript, page 70, lines 371-376): The text refers to the paper by Shaker et al. (reference #26) as if there were more than one study: “Cook’s distance analysis pinpointed 371 Shaker et al. (miR-20b and miR-17-3p)(26) as influential studies (Figure 7G). Yet, removing these studies caused only marginal changes: pooled sensitivity declined from 81.0% to 78.9%, and specificity increased from 80.0% to 81.1%. Interestingly, heterogeneity (I²) rose notably from 74.12% to 97.71%, suggesting that these studies contributed to controlled variance across studies. Outlier assessment confirmed no excessive deviations (Figure 7H).” Is it correct (“two studies”)?**

We appreciate this observation and agree that our wording could be misread. Shaker et al. is a single article, but it reports two distinct index tests (miR-20b and miR-17-3p), each with its own diagnostic accuracy metrics. In diagnostic test accuracy meta-analyses, when a paper evaluates multiple biomarkers with separate accuracy data, each biomarker is extracted as an independent DTA entry to avoid aggregation bias. This is reflected in Table 1, where certain authors appear more than once because different miRNAs were assessed, each contributing a separate 2×2 dataset.

To eliminate ambiguity, we have revised the Results wording as follows: *“Cook’s distance analysis identified two influential miRNA-specific entries from Shaker et al. (miR-20b and miR-17-3p) (26) (Fig. 7G). Removing these two miRNAs led to only marginal changes: pooled sensitivity declined from 81.0% to 78.9% and specificity increased from 80.0% to 81.1%; I² rose from 74.12% to 97.71%, indicating these entries contributed to controlled between-study variance. Outlier assessment confirmed no excessive deviations (Fig. 7H).”*

8. **Regarding I² (heterogeneity estimates), is it really necessary to use two decimal places to express this metric (Results section)?**

This point is well taken. To ensure consistent, reader-friendly formatting, we now report I² as an integer percentage (no decimals) throughout the manuscript. For the same reason, summary sensitivity and specificity are presented as whole percentages, with the accompanying 95% CIs providing the formal measure of precision. Readers who need finer granularity can consult the forest plots/SROC panels and the supplementary dataset, where the underlying exact values are available.

In addition, to avoid redundancy and reduce visual clutter in the subgroup Results text, we have removed inline 95% confidence intervals for subgroup metrics (sensitivity, specificity, DOR, etc.). These CIs are reported consistently and in full in the corresponding tables (Tables 2–4) and the Supplementary dataset, which serve as the canonical source for detailed numerical results. This change streamlines the narrative while preserving all necessary precision for interested readers.

Also, We have added a statement to the Statistical Analysis section clarifying that percentages are rounded for clarity and consistency. The text reads:

*“Percentages (e.g., sensitivity, specificity, and I²) are reported as whole numbers rounded to the nearest integer; exact estimates and 95% confidence intervals are provided in the corresponding tables and figures.”*

9. **Limitations section (pdf file, revised manuscript, page 75, lines 481-483):“Fourth, pre-analytical and analytical heterogeneity was substantial across studies. Variations in the biological sample type, normalization strategies, and miRNA isolation and detection platforms contributed significantly to inconsistency in results.” It is known that these factors affect the quantification of miRNA expression. But are the authors referring to the results of their analyses? Does “detection platforms” mean the method of quantifying microRNAs? I did not find any mention to the miRNA isolation method used in the previous studies in the manuscript. Moreover, as per Table 1 and S3 Table, all but one study measured the miRNA levels by RT-qPCR. In sum, did the authors perform the meta-analysis considering these two factors (miRNA isolation and detection platforms)?**

Thank you for raising this point. We confirm that the limitation refers to our own findings and to the variables we analyzed/explored for heterogeneity.

- What “detection platforms” means: it denotes the miRNA quantification method (e.g., RT-qPCR vs RNA-seq). In our dataset, 15/16 studies used RT-qPCR and 1 used RNA-seq (Table 1). Because of this near-absence of contrast, platform could not be modeled as a moderator without producing unstable estimates; hence it is discussed as a potential source of heterogeneity and listed as a limitation.
- What we did analyze: we pre-specified and modeled specimen type and normalization strategy, and they indeed showed effects in subgroup analyses and/or meta-regressions (Tables 2–4; Fig. 6). For example, normalization strategy was significantly associated with sensitivity in all three comparisons in the meta-regression, and specimen type was relevant in several models. These are precisely the kinds of analytical factors we call out in the Limitations.
- Isolation method (extraction kits/protocols): reporting was inconsistent across primary studies, which prevented reliable coding and formal subgroup/meta-regression by isolation method. We therefore treat isolation as a plausible pre-analytical contributor to between-study variability, supported by methodological literature cited in our Discussion (e.g., effects of matrix, processing delays, and extraction workflows on circulating miRNA measurements).
- Why normalization can drive heterogeneity (illustrative example): if Study A normalizes plasma miRNAs to U6 (which may be unstable in cell-free matrices) and Study B to miR-16-5p, the ΔCq baseline shifts, altering fold-change, implicit thresholds, and thus the 2×2 counts at a given cut-point—even when underlying biology is similar. This yields different sensitivity/specificity estimates and inflates heterogeneity unless normalization is harmonized or modeled—as we attempt to do via subgroup/meta-regression on normalization strategy

10. **Editing and typo errors:**
a) **Abstract (pdf file, page 53, lines 20-23): “We searched PubMed, CENTRAL, Scopus, Web of Science, ScienceDirect and ClinicalTrials (up to 15 January 2025) for diagnostic test accuracy studies of circulating miRNAs in three clearly defined groups: […]”. However, in the Methods section, “Search strategy” subsection (page 57, lines 114-116), it is stated that the last update of this review was on January 20, 2025. Which date is correct?**

Thank you for noting this inconsistency. The correct final search date is 20 January 2025. The mention of 15 January 2025 in the Abstract was a drafting error. We have harmonized the manuscript accordingly:

•	Abstract: “…searched PubMed, CENTRAL, Scopus, Web of Science, ScienceDirect, and ClinicalTrials through 20 January 2025 …”

•	Methods – Search strategy: “The last search was run on 20 January 2025.”

For transparency, the appended S1 Text (Complete Search Strategy) also documents 20 January 2025 as the final search date. This correction does not affect study inclusion, analyses, or conclusions.

b) **Percentages (sensitivity and specificity) remain formatted with a space between the numeral and the percentage symbol in the Abstract section (pdf file, page 54)**

Thank you for pointing this out. We have corrected the formatting in the Abstract to remove the space before the percent symbol (e.g., “80%” instead of “80 %”) and have standardized this convention throughout the manuscript for consistency.

c) **Reviewer 4 states in comment #1.4: “lines like “overall evidence certainly was graded via GRADE” (line 26) sound off. Perhaps intended “certainty was graded”; a correction improves precision.” In the rebuttal letter, the authors responded that the abstract has been revised to state “overall evidence certainty was graded via GRADE”. The word “certainly” was removed, but “certainty” was not added. Thus, the revised sentence states that “[…] overall evidence was graded via GRADE.” (pdf file, page 53, line 29).**

You are right, this was an oversight on our part. We have corrected the Abstract to align with GRADE terminology:

Revised Abstract wording:

“Two reviewers independently performed study selection, data extraction and risk-of-bias assessment using QUADAS-2; the certainty of the evidence was assessed using GRADE.”

d) **NLR is written as NPL in the caption of Figure 4 (pdf file, page 66, line 302).**

Thank you for catching this typographical error. We have corrected “NPL” → “NLR” in the Figure 4 caption and verified consistency across the manuscript (text, tables, and figures) for positive (PLR) and negative (NLR) likelihood ratios.

e) **Results section (pdf file, page 69, lines 357-358): “PDR” is spelled as “DR”.**

Thank you for catching this typographical error. We have corrected “DR” → “PDR” in the Results and verified consistency across the manuscript, including figures and tables.

f) **In the caption of Figure 7 (pdf file, page 70, lines 385-386), the Fig. 7E refers to both the goodness-of-fit and the bivariate normality, while the Fig. 7B does not exist: “Figure 7. Sensitivity analysis. Goodness-of-fit (A,E,I), Bivariate normality (E,F,J) Influence analysis (C,G,K) and Outlier detection (D,H,L) across: CTL vs DR, T2DM vs DR and NPDR vs PDR.”**

We thank the reviewer for catching this captioning error. Panel B was incorrectly omitted from the “Bivariate normality” list (and E was duplicated). We have corrected the caption to “Bivariate normality (B, F, J)” and verified alignment between panels and in-text references.

g) **Discussion section (pdf file, page 72, lines 416-418): The first paragraph ends with a sentence with missing terms: “Furthermore, the study lacks a formal assessment of risk of bias using tools such as QUADAS-2, nor does it explore heterogeneity or undermining both its methodological robustness and reproducibility.” Explore heterogeneity and what else?**

We agree that this sentence was ambiguously phrased. Our intent was to state that the prior review (Zhou et al., 2020) did not explore heterogeneity and that this thereby undermines methodological robustness and reproducibility. We have corrected the sentence to:

*“Furthermore, the study lacks a formal risk-of-bias assessment (e.g., QUADAS-2) and does not explore heterogeneity, thereby undermining both its methodological robustness and reproducibility.”*

h) **Discussion section (pdf file, page 73, lines 426-430): “By stratifying the analysis into three distinct and clinically relevant comparison groups—healthy controls CTL vs DR, T2DM , T2DM vs DR, and NPDR vs PDR, we were able to reduce inter-study heterogeneity and increase the precision and relevance of pooled diagnostic accuracy estimates comparing with Ma et al (2022).” The term “T2DM” before “T2DM vs DR” should be removed.**

Thank you for noting this. We have removed the extraneous “T2DM.

i) **Discussion section (pdf file, page 73, lines 439-441): The following sentence needs to be reworded for clarity and completeness: “miR-21, also was found in 3 studies (15, 18, 34) has been implicated in retinal angiogenesis and inflammation in the diabetic context (38).**

Thank you for this suggestion. We have revised the sentence for clarity and grammatical correctness as follows:*“miR-21 was also reported in three studies (15, 18, 34) and has been implicated in retinal angiogenesis and inflammation in diabetes (38).*

j) **“Prisma” is spelled as “Prima” in the title of S2 Table (pdf file, page 80, line 603).**

Thank you for noting this. We have corrected the title to “PRISMA” in S2 Table and verified consistency across the manuscript and Supplementary materials.

k) **S4 Table is numbered as S5 Table in the manuscript (pdf file, page 80, line 605).**

Thank you for noting this. We have corrected the label to S4 Table and updated all corresponding in-text references and cross-references to ensure consistency.

l) **Should not supplementary tables be mentioned in the text (accompanying the respective methods or results)?**

We agree. We have added an explicit in Methods (Data extraction) and (Statistical analysis) The sentences now reads:

“From each included study, we extracted data on study characteristics (author, year, country, design), population details, biological sample type, index test platform, normalization strategy, and diagnostic accuracy metrics (sensitivity, specificity, and AUC). The complete extraction dataset is provided in S3 Table.”

AND

“All statistical analyses were performed at 95% CI, using STATA 18 (STATA Corporation, College Station, TX, USA), incorporating the MIDAS package for meta-analysis of DTA. Additional details are provided in S4, S5, and S6 tables.”

m) **S3 Table (“Extraction data”) presents some Spanish words (“Casos y controles”, “Controles”, “Egipto”, “Cuantitative”, and “Cualitative”). Moreover, a term is missing from the “Controls (Healthy and T2DM)” column specifying who the 70 control subjects are in the study by Wan et al., 2017.**

Thank you for noting these issues. We have corrected all non-English entries in S3 Table, replacing them with their English equivalents (“Cases and controls”, “Case-Controls”, “Egypt”, “Quantitative”, “Qualitative”) and verified consistent terminology across the table. In addition, we have completed the “Controls” field for Wan et al., 2017.

n) **Bibliography (pdf file, page 83, lines 769-772): The text citing the study by Théry et al. (reference #55) was both added and removed during the manuscript review, but the reference remains in the Bibliography.**

Thank you for pointing this out. The citation to Théry et al. (former ref.#55) was inadvertently retained in the bibliography after the corresponding in-text mention was removed. We have deleted this reference from the Bibliography and verified that no residual in-text citations to this source remain.
