# Supplementary material for: Circulating microRNAs as biomarkers for diabetic retinopathy stage identification: A DTA systematic review and meta-analysis
Source: PLoS One. 2025 Nov 21;20(11):e0335434. doi: 10.1371/journal.pone.0335434 (PMC12637958; doi:10.1371/journal.pone.0335434)
Supplement: Attachment — Submitted filename: Response to Reviewers.docx [file pone.0335434.s005.docx]

**REBUTTAL LETTER (Reviewer 5)**

**Reviewer #5:** The manuscript was revised to address the comments raised in the previous submission (R1). However, the submitted manuscript (R2) does not present some of the modifications stated in the rebuttal letter and some errors (that did not exist) were introduced into the new version of the manuscript (PONE-D-25-27197_R2.pdf file). After I started writing my comments, I found out that the “clean manuscript” (pages 15 to 39) is not identical to the “manuscript with track changes” (pages 48 to 73), which makes it difficult to follow what has been modified (or not) in the revised manuscript. The comments I provide below are based on the manuscript included on pages 48 to 78. Finally, I have to say that I did not check the figures and the supplementary tables in the revised manuscript.

**1) Abstract section (revised manuscript with track changes, page 48): In the response to the comment #1a, the authors state in the rebuttal letter that they “have revised the first sentence of the Abstract accordingly to improve clarity and avoid redundancy. The updated sentence now reads: “Purpose: To evaluate the diagnostic accuracy of circulating miRNAs in distinguishing between different diabetic retinopathy stages in type 2 diabetes mellitus.” Although this detail does not compromise the manuscript, the purpose remains the same. Instead, the second sentence (first one of the Methods) was modified (revised manuscript with track changes, page 48, lines 18-20).**

We appreciate your observation. Upon re-evaluating the Purpose and Methods sections, we noticed that they could lead to ambiguity due to a repetition of the study objective. Accordingly, we have reformulated both sections to avoid redundancy and improve clarity in abstract section.

**2) Abstract section (revised manuscript with track changes, page 48): An error was introduced in the sentence “We searched PubMed, CENTRAL, Scopus, Web of Science, ScienceDirect and ClinicalTrials (up to 20 January 2025) for diagnostic test accuracy studies […]” (lines 21-27) by deleting the following terms: “type 2 diabetes mellitus without retinopathy versus diabetic retinopathy”. Now, the sentence is incomplete.**

We understand the reviewer’s concern. However, we would like to clarify that this was an intentional choice to avoid redundancy. In the Abstract (lines 17–18), we had already introduced the full terms and abbreviations for diabetic retinopathy (DR) and type 2 diabetes mellitus (T2DM). Repeating these abbreviations immediately in the following sentence would unnecessarily lengthen the Abstract without adding clarity. By maintaining the current formulation, the meaning remains fully understandable while ensuring conciseness, which is essential for the Abstract**.**

**3) Introduction section (revised manuscript with track changes, page 49):**

**In response to the comment #2, the reference by Lundeen et al. was replaced by the paper by Thomas et al. However, the study by Lundeen et al. remains in the Bibliography (page 70) as the reference #2. Therefore, all references from Th**

We appreciate the reviewer’s observation. The discrepancy arose because the EndNote bibliography was not fully updated in the track-changes version of the manuscript. However, the correction was made in the clean version, where the reference list has been properly updated and renumbered. We regret the oversight and confirm that the final revised manuscript reflects the correct reference Thomas et al. onward should be renumbered.

**4) Results section (revised manuscript with track changes, page 56): In response to the comment #5, the authors added a column with the total N in Table 1 described on page 22, but not on that presented on page 56. I think this is not a problem, as the Journal uses the clean version of the manuscript to typeset the proof.**

Thank you for this observation. We had not realized that while the updated version of Table 1 (including the total N column) was uploaded as an independent file, the same correction was not applied in the manuscript version. We have now corrected this inconsistency and ensured that the revised manuscript includes the updated Table 1

**5) Results section (revised manuscript with track changes, page 57): In response to the comment #8, the authors decided to report I² as an integer percentage (throughout the manuscript). However, the following sentence still has the I² estimates with two decimals between the parentheses and needs to be corrected (lines 203-206): “The pooled estimates from the random‐effects model showed a summary sensitivity of 77% (70–82), with an I² of 47% (1.39–92.75), and a summary specificity of 84% (77–89), with an I² of 62% (31.26–93.30), indicating moderate heterogeneity (Fig 3A-B).”**

We agree with the reviewer’s observation and realized that in some parts of the manuscript we reported the confidence intervals for heterogeneity (I²), while in others we did not. To avoid confusion, we have removed the confidence intervals for I², as the most relevant information lies in reporting the confidence intervals for sensitivity and specificity. Moreover, the forest plot figures already provide the confidence intervals for I², should readers wish to consult them. We have applied the same approach to the reporting of confidence intervals for sensitivity, specificity, and DOR in the section Subgroup Analysis and Meta-Regression in CTL vs DR, T2DM vs DR, and NPDR vs PDR Comparisons. There are three tables that clearly present all confidence intervals in detail. Including excessive numerical information in the text may hinder the readability of the results. Furthermore, we have observed that other scientific articles follow a similar approach in order to highlight their key findings in the subgroup analyses.

**6) Editing and typo errors: In response to the comment #10m, the authors added the following sentences to the Methods section: “The complete extraction dataset is provided in S3 Table.” and “Additional details are provided in S4, S5, and S6 tables.” These additions were made to the clean manuscript (pages 19 and 21), but not to the manuscript with track changes.**

We regret the confusion. These sentences have now been properly added in the revised manuscript with track changes, so that both versions are consistent.

**7) Funding section: In the response to the comment #1b, the authors state in the rebuttal letter that “no funds from these grants were specifically allocated or used for the conduct of this systematic review and meta-analysis. To avoid any possible misunderstanding, we have removed the Funding section from the manuscript and clarified that this work received no dedicated external financial support.” However, the Funding section was neither removed from the manuscript nor corrected according to this statement (revised manuscript with track changes, page 69).**

The reviewer is correct, and we apologize for this misunderstanding. Since several authors were revising the manuscript simultaneously, an earlier version was mistakenly used as the basis for further edits, which led to the Funding section not being updated as intended. This error has now been corrected, and the revised manuscript accurately reflects the statement provided in our rebuttal.
